# Computational prediction of muscle synergy using a finite element framework for a musculoskeletal model on lower limb

**DOI:** 10.3389/fbioe.2023.1130219

**Published:** 2023-07-18

**Authors:** Sentong Wang, Kazunori Hase, Tetsuro Funato

**Affiliations:** ^1^ Graduate School of Informatics and Engineering, The University of Electro-Communications, Tokyo, Japan; ^2^ Graduate School of Systems Design, Tokyo Metropolitan University, Tokyo, Japan; ^3^ Faculty of Systems Design, Tokyo Metropolitan University, Tokyo, Japan

**Keywords:** muscle synergy, muscle activity, non-negative matrix factorization, musculoskeletal model, knee

## Abstract

Previous studies have demonstrated that the central nervous system activates muscles in module patterns to reduce the complexity needed to control each muscle while producing a movement, which is referred to as muscle synergy. In previous musculoskeletal modeling-based muscle synergy analysis studies, as a result of simplification of the joints, a conventional rigid-body link musculoskeletal model failed to represent the physiological interactions of muscle activation and joint kinematics. However, the interaction between the muscle level and joint level that exists *in vivo* is an important relationship that influences the biomechanics and neurophysiology of the musculoskeletal system. In the present, a lower limb musculoskeletal model coupling a detailed representation of a joint including complex contact behavior and material representations was used for muscle synergy analysis using a decomposition method of non-negative matrix factorization (NMF). The complexity of the representation of a joint in a musculoskeletal system allows for the investigation of the physiological interactions *in vivo* on the musculoskeletal system, thereby facilitating the decomposition of the muscle synergy. Results indicated that, the activities of the 20 muscles on the lower limb during the stance phase of gait could be controlled by three muscle synergies, and total variance accounted for by synergies was 86.42%. The characterization of muscle synergy and musculoskeletal biomechanics is consistent with the results, thus explaining the formational mechanism of lower limb motions during gait through the reduction of the dimensions of control issues by muscle synergy and the central nervous system.

## 1 Introduction

Despite decades of research, there remains uncertainty about how the human central nervous system coordinates the activity of large numbers of muscles during motion (Rabbi et al., 2020). Many studies have shown that the central nervous system activates muscles in groups to reduce the complexity needed to control each muscle while performing one movement (d’Avella et al., 2003; Ivanenko et al., 2004). The coordination of synchronously activated muscles is often referred to as muscle synergy (Lee, 1984). Circumstantial evidence suggests that muscle synergy occurs in the brain stem and/or spinal cord and follows a modular organization (Tresch and Bizzi, 1999). Understanding the organization of muscle synergy may help elucidate the neural mechanisms that underlie a variety of neurological disorders, including stroke (Pan et al., 2018; Funato et al., 2022), cerebral palsy (Goudriaan et al., 2022), and Parkinson’s disease (Falaki et al., 2016).

The concept that underpins the essence of muscular synergy analysis is dimensionality reduction. Muscle synergy analysis assumes that signals of muscle activity are not independent of each other but can be decomposed into a smaller number of independent control signals known as neural controls. In muscle synergy analysis, applying a decomposition approach to a set of muscle activation schemes involves two components: muscle synergy and activating coefficients. The muscle synergies scale the neural control intensity of excitation primitives to represent time-varying neural controls (Lambert-Shirzad and Van der Loos, 2017). For gait motion, only three to five muscle synergies are typically needed to represent over 90% of the variability in many muscle activities of the lower limbs (Ivanenko et al., 2004; Cappellini et al., 2006; Clark et al., 2010). In recent years, a decomposition method of non-negative matrix factorization (NMF) for muscle synergy analysis has become increasingly popular (Paatero et al., 1991; Paatero and Tapper, 1994; Lee and Seung, 1999). NMF is a non-linear decomposition technique that has the property of having no negative elements and outputs the optimized basis (synergy) vectors and corresponding weight (coefficient) vectors by minimizing the error between the original signals and the reconstructed data. NMF has been used by many researchers to separate neural control from signals of muscle activity in humans (Rabbi et al., 2020) or animals (Huffmaster et al., 2018). The reliability of muscle synergy prediction is entirely dependent on the quality of obtained muscle activities. Surface electromyography (EMG) recording is non-invasive and easy to apply, and has been the most popular method for measuring muscle electric activity in biomechanical research. However, surface EMG measurement has a practical challenge in its inability to acquire a signal from deep muscles (Sartori et al., 2014; Zonnino and Sergi, 2019). For example, it is practically impossible to collect muscle electrical activity signals from deep femur muscles, such as the vastus intermedius, and hip muscles, such as the iliacus and psoas, using surface EMG. When EMG data from important deep muscles are missing from the EMG measurement, force estimates for other muscles with similar roles may be significantly overestimated (Zonnino and Sergi, 2019), even though recently some hybrid musculoskeletal modeling studies have been partially able to address the issue of missing EMGs (Sartori et al., 2014; Ao et al., 2022). In addition, EMG data have inherent potential challenges that may limit the accuracy of estimating muscle activity, such as noisy signals from crosstalk between adjacent muscles (Germer et al., 2021), motion artifacts (Luca et al., 2010), and difficulty in obtaining true maximal muscle excitation for EMG normalization (Hodder and Keir, 2013). Since EMG measurement cannot accurately obtain the activity of each muscle of the lower limbs, the reliability of muscle synergy estimation cannot be guaranteed using EMG data.

The comprehensiveness of muscle representation and the accuracy of muscle activity prediction determine the accuracy of muscle synergy. Currently, a computational approach, musculoskeletal modeling based on motion measurement data, has been proven to provide relatively accurate prediction of muscle activities on the whole body of a human (Rajagopal et al., 2016; Trinler et al., 2019). A static optimization algorithm is generally used to solve the muscle redundancy problem to estimate muscle activities in the whole body or in a local area of the body. The key to accurate prediction of muscle activities using static optimization is the ability to represent the physiological interaction between joint kinematics and muscle dynamics. The muscle activities contribute to the joint kinematics and could thus change the deformation of the cartilage soft tissue. The deformed soft tissues could change the joint secondary kinematics, which in turn would seriously affect the muscle length and muscle moment arm, thus affecting the estimation of the muscle activities. However, traditional musculoskeletal models, such as OpenSim (Delp et al., 2007) and AnyBody (Kang et al., 2019), have been mostly constructed in multi-body dynamics with the skeletal structure being modeled as a rigid body and the joint being modeled as a simple specific degree-of-freedom rotational joint. This provides no insight into the interaction between the joint kinematics and muscle activities, and the interaction is key to the representation of realistic muscle activity. Recent research has introduced musculoskeletal modeling on a finite element framework, combining musculoskeletal modeling and detailed soft-tissue deformation finite element analysis into a single framework (Shu et al., 2018; Wang et al., 2021a), which can estimate muscle activities while maintaining a realistic representation of the joint tissue deformation to provide insight into the realistic interaction between the joint kinematics and muscle activities. Furthermore, the muscle-skeletal model based on EMG has been extensively studied by many researchers (Sartori et al., 2012; Sartori et al., 2014; Davico et al., 2022; Esrafilian et al., 2022). The advantage of this model is that it can be directly driven by measured muscle activity. However, in our research, the representation of the secondary kinematics of the knee joint requires the participation of all muscles surrounding the knee joint, and the deep muscle activity is challenging to measure using EMG.

The purposes of the present study were 1) to demonstrate that joint tissue deformation affects muscle activity estimates when included in a musculoskeletal model and to verify that the muscle activities obtained with the proposed approach are consistent with the measured EMG, and are therefore plausible, and 2) to predict muscle synergies extracted using NMF from muscle activities estimated by static optimization during the stance phase of gait. A musculoskeletal model using a single concurrent framework combining the entire lower limb musculoskeletal dynamics and knee-joint finite element analysis was used (Wang et al., 2021b; Wang et al., 2022). The present study may provide a potential method to investigate the interrelationship between the nervous system and the joint system, such as the formational mechanism of joint constriction due to cerebral palsy.

## 2 Methods

### 2.1 Subject experiments

A healthy male participant (age: 22 years, height: 170 cm, weight: 60.8 kg) participated in the gait measurement. The participant was thoroughly informed about the purpose, methods, and caveats of the experiment. The experiment was approved by the research ethics committee of Doshisha University. The kinematic, ground reaction force and muscle EMG data were collected from the participant as he walked on a dual-belt treadmill instrumented with two force plates for 60 s at a self-selected pace (1.3 m/s) with his preferred gait during the experiment. The marker-based motion trajectories (sampling frequency: 500 Hz, MAC3D Digital RealTime System, Motion Analysis Corp, UK) were used to compute the positions of 14 optical markers, and the ground reaction force (sampling frequency: 500 Hz, Bertec, Columbus, OH, United States of America) was synchronously collected during walking at the preferred gait. In addition, EMG signals were recorded from 12 muscles on the right lower limb at 1,000 Hz (Biolog DL-3100, S&ME Corp, US; WEB-7000, Nihon Kohden, JP; FreeEMG, BTS Bioengineering, ITA). For all EMG signals, high-pass filtering was performed using a Butterworth filter with a cut-off frequency of 1 Hz, and the signals were demeaned, rectified, and low-pass filtered using a Butterworth filter with a cut-off of 5 Hz. Finally, the normalized peak EMG activity for each muscle was obtained.

### 2.2 Finite element musculoskeletal model

**FIGURE 1:**
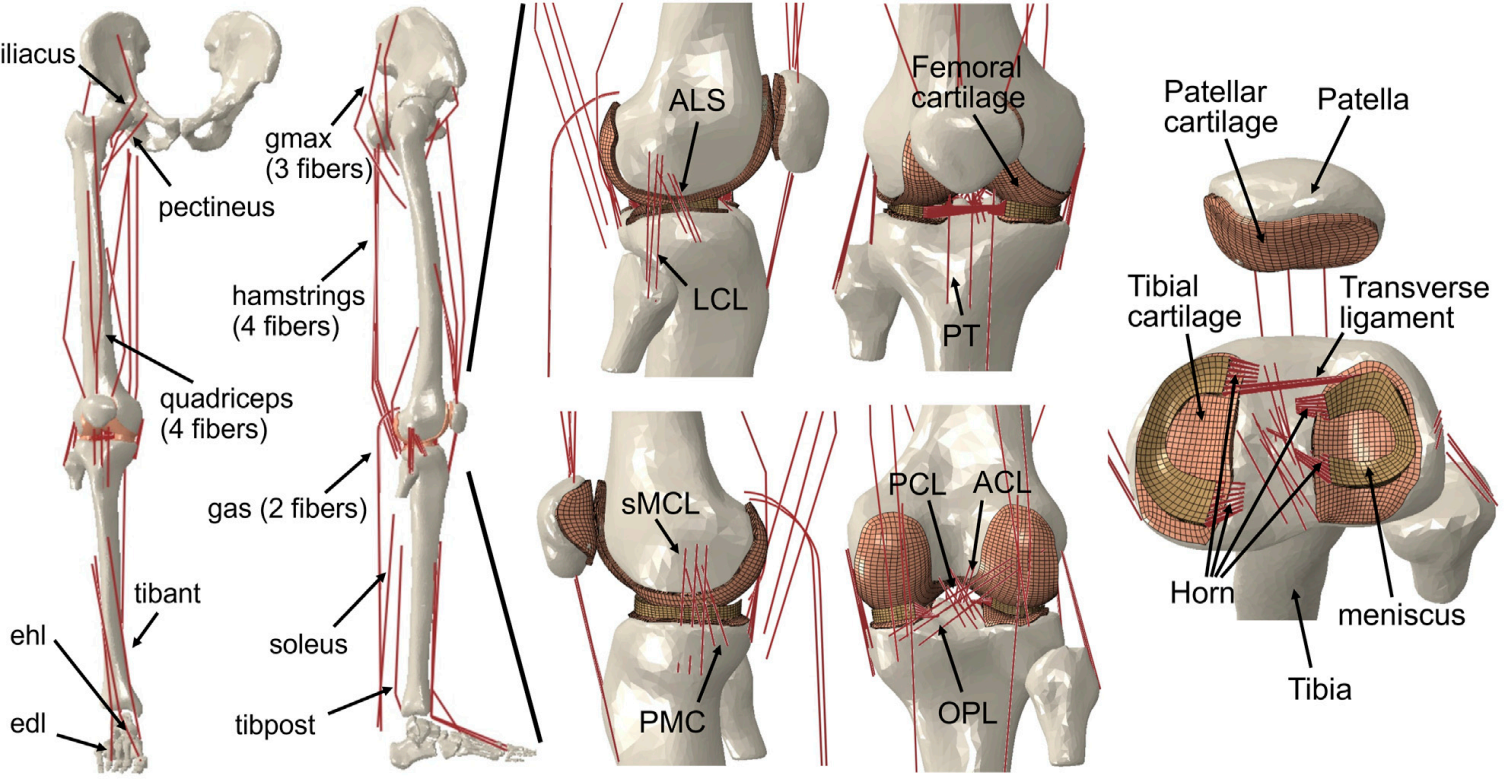
Right lower limb finite element framework for musculoskeletal model containing knees. The musculoskeletal model includes a 12-DOF knee joint, along with 20 muscles (gluteus maximus [gmax]; iliacus; pectineus; quadriceps: rectus femoris, vastus medialis, vastus intermedius, vastus lateralis; hamstrings: semimembranosus, semitendinosus, biceps femoris; gastrocnemius [gas]; soleus; tibialis anterior [tibant], tibialis posterior [tibpost]; extensor halluces longus [ehl]; and extensor digitorum longus [edl]); ligaments (anterior cruciate ligament [ACL], posterior cruciate ligament [PCL], medial collateral ligament [MCL], posteromedial capsule [PMC], lateral collateral ligament [LCL], anterolateral structure [ALS], oblique popliteal ligament [OPL], and transverse ligament); cartilage: the meniscus; and the meniscus horn attachments.

In the present study, a musculoskeletal model of the right lower limb was developed using a finite element framework, specifically the ABAQUS/Explicit (SIMULIA, Providence, RI, United States) as shown in Figure 1. The modeling approach of the finite element musculoskeletal (FEMS) model of the lower limb included a healthy knee, which was described in detail in previous studies by Wang et al. (2021a) and Wang et al. (2022). However, a summary of the modeling approach is provided below. In order to construct the FEMS model, the geometries of the bones in the right lower limb, as well as the articular cartilages and meniscus in the knee, were obtained from computed tomography and magnetic resonance imaging scans. These structures were manually segmented and reconstructed for modeling purposes. The femoral, tibial, and patellar bones were represented using rigid triangular shell elements, while the cartilage and meniscus were meshed using elastic eight-node hexahedral elements. The articular cartilages were assumed to exhibit linear elastic isotropic behavior based on the work of Li et al. (2001). The menisci were defined to be transversely isotropic (Donahue et al., 2002; Yao et al., 2006), following studies by Donahue et al. (2002) and Yao et al. (2006). Contact behaviors between different tissue pairs within the knee were defined, incorporating a coefficient of friction of 0.04 based on the findings of McCann et al. (2009). The model incorporated various joint types to represent the different articulations of the lower limb. A spherical joint with three degrees of freedom (DOFs) was used to describe the hip joint. The tibiofemoral and patellofemoral joints, which allow for six DOFs, were represented using appropriate joint elements. Finally, a hinge joint at the ankle, with one DOF, was included in the model. In order to simulate the behavior of ligaments in the knee joint, non-linear spring bundles were used. The anterior cruciate ligament (ACL), posterior cruciate ligament (PCL), medial collateral ligament (MCL), posteromedial capsule (PMC), lateral collateral ligament (LCL), anterolateral structure (ALS), and oblique popliteal ligament (OPL) were all modeled using force-strain relationships, as proposed by Abdel-Rahman and Hefzy in 1998:

$$\begin{array}{c} f_{i}=\left\{\begin{array}{cc} 0 & \varepsilon_{i}\leq 0\\ k_{i}^{1}{\left(l_{i}-l_{i}^{0}\right)}^{2} & 0<\varepsilon_{i}\leq 2\varepsilon^{l}\\ k_{i}^{2}\left[l_{i}-\left(1+\varepsilon^{l}\right)l_{i}^{0}\right] & 2\varepsilon^{l}\;<\varepsilon_{i} \end{array}\right. \end{array}$$ (1)

where $l_{i}$ is the current length in the i th ligament, $l_{i}^{0}$ is the slack length of the i th ligament, $\varepsilon^{l}$ is the strain (assumed to be constant at 0.03), and $k_{i}^{1}$ and $k_{i}^{2}$ are the stiffness coefficients of the spring elements representing the i th ligament for the non-linear toe and linear regions, respectively. The values of the material properties of the non-linear spring elements are listed in Table 1 (Abdel-Rahman and Hefzy, 1998; Yu et al., 2001).

**TABLE 1:** Stiffness parameters of ligaments.

Ligament	Ligament bundle	Number of bundles	$k^{1}$ ( $N\;{mm}^{-2}$ )	$k^{2}$ ( $N\;{mm}^{-1}$ )
ACL	anterior	2	22.48	83.15
	posterior	2	26.27	83.15
PCL	anterior	2	31.26	125.00
	posterior	2	19.29	60.00
MCL	anterior	1	10.00	91.25
	oblique	1	5.00	27.86
	deep	1	5.00	21.07
PMC	—	3	12.00	52.59
LCL	—	3	10.00	72.22
ALS	—	3	5.00	19.00
OPL	—	3	3.00	21.42

Modeling of the anterior transverse ligament, which connects the anterior convex margin of the lateral meniscus to the anterior end of the medial meniscus, was performed using multiple linear spring elements. The stiffness of these spring elements was set to 12.5 N/mm, as determined by Donahue et al. (2003). Regarding the four meniscal horn attachments, they were also represented as multiple linear spring elements. The stiffness constants for these springs were calculated based on the reported Young’s modulus for the horn attachments, as investigated by Hauch et al. (2009). These spring elements were used to firmly connect the faces of the meniscal horns to the tibial bone, as depicted in Figure 1.

The FEMS model incorporated a total of 20 representative linear element muscles that are present in the lower limb. These muscles included the following: gluteus maximus (three units), iliacus, pectineus, rectus femoris, vastus medialis, vastus intermedius, vastus lateralis, semimembranosus, semitendinosus, biceps femoris (short and long heads), gastrocnemius (medial and lateral heads), soleus, tibialis (anterior and posterior), extensor hallucis longus, and extensor digitorum longus. The mechanical properties of these muscles were characterized using a Hill-type model, as described by Zajac (1989). This model consists of three main components arranged in series and parallel configurations. The contractile element represents the active fiber force-length property of the muscle, the passive elastic element represents the passive fiber force-length property, and the elastic tendon element is included in series with the other elements.

The musculotendon parameters (e.g., optimal force, optimal fiber, tendon slack length, and pennation angle) are based on studies of cadavers (Rajagopal et al., 2016), and the scaled optimal fiber and tendon slack length of each muscle was adjusted to ensure the consistency of the force-length relationship of the muscle model (Arnold and Delp, 2011). The wrapping between the muscles and femoral and tibial bones was considered in the model to approximate the muscle paths using some wrapping surface (Ali et al., 2016) and dashpot element.

### 2.3 Static optimization for muscle activations

**FIGURE 2:**
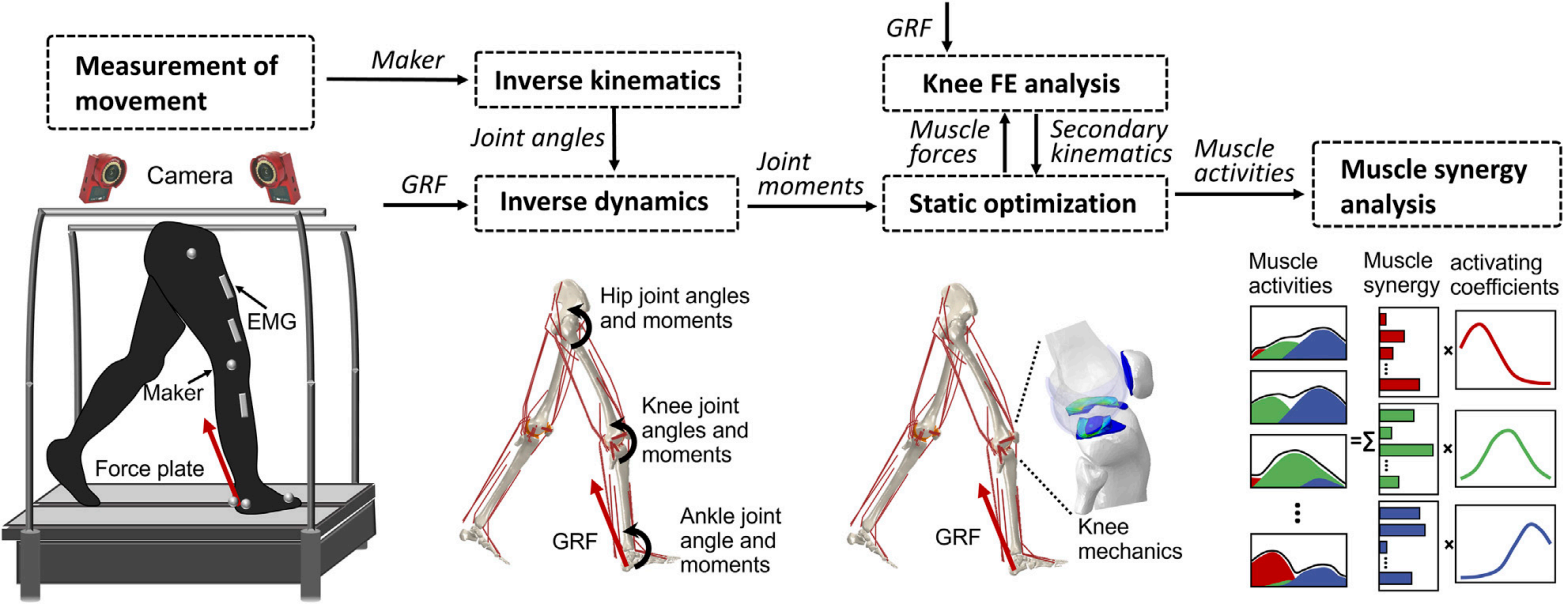
Overview of the computational muscle synergy analysis. Marker positions were input into the inverse kinematics to predict the joint primary angles (lumbar flexion–extension, hip joint 3–DOF rotations, knee joint flexion–extension, and ankle dorsiflexion–plantarflexion). The joint primary angles on each joint and the ground reaction force measured from the force plate were input into the inverse dynamics in order to predict the muscle lengths, muscle moment arms, and right lower limb joint moments. The static optimization of the muscle force was performed with the joint moments, the muscle lengths, and the muscle moment arms as inputs to predict the muscle force. The knee finite element analysis was performed from the muscle forces and the ground reaction force to predict the knee joint secondary kinematics and contact mechanics. The predicted knee secondary kinematics was fed back to a new static optimization, and the muscle force optimization and knee contact mechanics were again computed. The muscle synergies were decomposed from the muscle activities using a method of non-negative matrix factorization.

Previous studies (Wang et al., 2021b; Wang et al., 2022; Wang et al., 2022) have provided a description of the gait analysis method that considers lower limb motion. In the following, a brief overview of the proposed method is given. The analysis incorporates inverse kinematics, inverse dynamics, muscle activity optimization, and FE analysis of the knee, using a motion capture system (Figure 2). The first step involves inverse kinematics analysis, which calculates the primary angles of joints (such as hip joint flexion–extension, internal–external rotation, abduction–adduction angles; knee joint flexion–extension angle; and ankle joint dorsiflexion–plantarflexion angle) based on marker trajectories obtained from the gait experiment. These primary joint angles, along with the ground reaction force measured by force plates, are then input into the inverse dynamics analysis. This analysis determines the joint moments and uses a model to calculate simultaneous muscle lengths, muscle moment arms, and joint axis vectors. Next, the muscle lengths, muscle moment arms, and joint axis vectors are fed into a static optimization algorithm. This algorithm estimates the muscle activities by satisfying an equilibrium equation of the joint moment. The muscle activities are optimized by minimizing the sum of the cube of the muscular activation $a_{m}$ , following the approach described by Hase and Yamazaki (2002):

$$\begin{array}{c} M_{j}={e_{j}}^{T}\left\{\sum_{m=1}^{20}\left[r_{jm}\left(\theta_{j},s\right)\times f_{jm}\left(a_{m}\right)\right]\right\} \end{array}$$ (2)

$$\begin{array}{c} I=\min\sum_{m=1}^{20}{a_{m}}^{3} \end{array}$$ (3)

where $M_{j}$ is the moment of the j th joint, $e_{j}$ is the unit vector along the rotation axes of the j th joint, $\theta_{j}$ is the primary angle of the j th joint, s is the secondary kinematics of the knee joint (please note that the hip and ankle joints only include primary angles), and $r_{jm}\left(\theta_{j},s\right)$ is the moment arm vector of the m th muscle for the j th joint. With each muscular activation considered as a variable, the force of the m th muscle can be expressed as a function $f_{jm}\left(a_{m}\right)$ of the activation about the j th joint. In addition, the muscular activations were constrained to be in the range of zero to one (inclusive).

The static optimization was implemented by subroutines written in MATLAB (R2018b, MathWorks, MA, United States) using the interior-point method at 16 evenly distributed time points while combining concurrent FE analysis of the knee joint in ABAQUS/Explicit during the stance phase of gait. The muscle forces obtained from the static optimization, along with the ground reaction force and ground reaction moment acting on the foot center of mass, were imposed as boundary conditions. Since the remaining DOFs except flexion–extension were left unconstrained, the knee joint secondary kinematics determined entirely from the interaction of the joint contact behavior, muscle forces, and ligament restraint. Subsequently, the computed knee secondary kinematics was fed back into a new static optimization step, resulting in updated muscle lengths and moment arms. This iterative process allowed for the re-estimation of muscle forces and the computation of knee secondary kinematics until the convergence criteria of static optimization for equilibrium in the joint moment were satisfied. In summary, each muscle activation static optimization takes into consideration the muscle lengths, muscle moment arms, and joint axis vectors of the knee joint determined by the current FE analysis. In order to investigate the influence of the complexity of the representation of the knee joint on muscle activity, we simultaneously constructed a rigid body musculoskeletal (MS) model and used static optimization to infer muscle activity. The model lacks knee joint tissue geometry and soft tissue contact behavior and is constrained to a flexion-extension 1–DOF hinge joint.

### 2.4 Muscle synergy analysis

Muscle synergies were extracted from the muscle activities, which were estimated by a concurrent simulation that performs the static optimization with the knee FE analysis. Muscle synergies were identified by NMF with the multiplicative update method (Lee and Seung, 1999; Funato et al., 2022). Matrix A ( t×m , where t is the number of samples and m is the number of muscles) is arranged so that each column is the time series of the muscle activity $a_{m}$ for each muscle, and NMF decomposes matrix A as the product of two matrices, one of which is represented as a time-invariant component (muscle synergy) W that co-activates multiple muscles, and the other of which is activated by a temporal waveform (activating coefficients) H :

$$\begin{array}{c} A=HW^{T}+r=\sum_{p=1}^{n}h_{p}{w_{p}}^{T}+r \end{array}$$ (4)

where $w_{p}$ is the component for the p th synergy, the columns of matrix W ( m×n , where n is the number of synergies) are the muscle synergies, $h_{p}$ is the temporal waveform for the p th synergy, the columns of matrix H ( t×n ) are their activations, r indicates the residuals, and A is the muscle activity. The above parameters have only non-negative values.

The “nnmf” function in MATLAB (R2021b, MathWorks, MA, United States of America) was adopted in order to compute NMF. The number of synergies was determined by setting the threshold of the variance accounted for (VAF) (Torres-Oviedo et al., 2006). We calculated the VAF for a single stance phase of gait and selected the minimum number of synergies with the VAF exceeding 80% as the number of synergies for the computational prediction (Torres-Oviedo et al., 2006; Tresch et al., 2006).

Using the same method, muscle synergies were decomposed from EMG data, while the muscle synergies obtained from EMG data were regularized based on each peak activating coefficient obtained from the model.

## 3 Results

**FIGURE 3:**
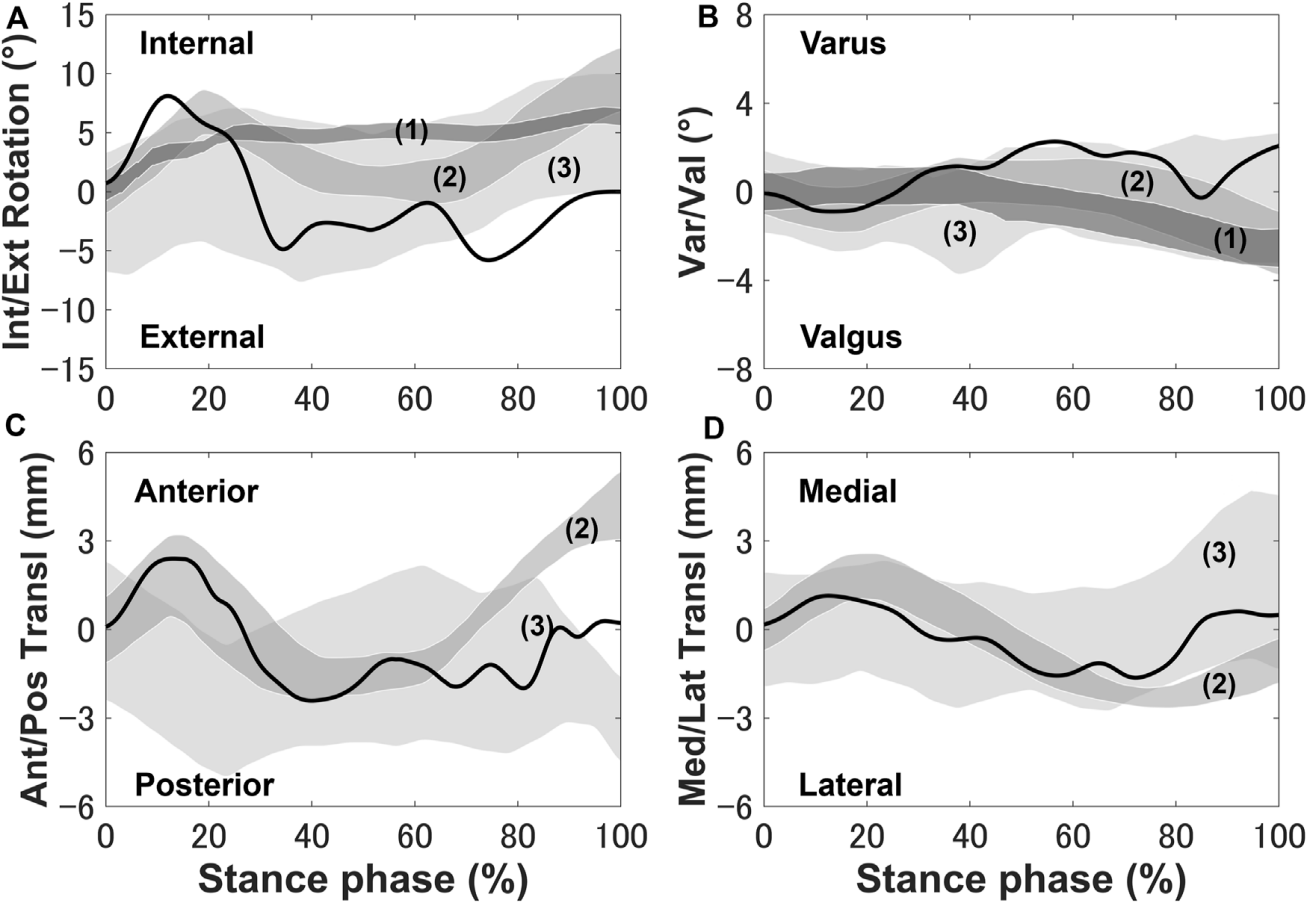
Knee secondary kinematics of the tibia with respect to the femur predicted using the model plotted and *in vivo* experimental results (shaded area) from previous studies: **(A)** internal–external rotation, **(B)** varus–valgus, **(C)** anterior–posterior translation, and **(D)** medial–lateral translation. The measurement data were obtained from (1) Gray et al. (2019), (2) Kozanek et al. (2009), and (3) Clément et al. (2018) and are shifted to start from zero to exclude any differences in the choice of coordinate system.

The tibia was internally rotated during the loading response, where the maximum internal rotation was 8.6°, and external rotation occurred where the peak external rotation reached 5.2° (Figure 3). Next, the tibia was placed in the external rotation position and underwent a slight rotation until the tibia returned to the initial angle at toe-off. The anterior translation of the tibia exhibited a 2.5 mm trend near the loading response, whereas the posterior translation of the tibia occurred, remained in the posterior translation position and a slight anterior translation occurred during the pre-swing phase.

**FIGURE 4:**
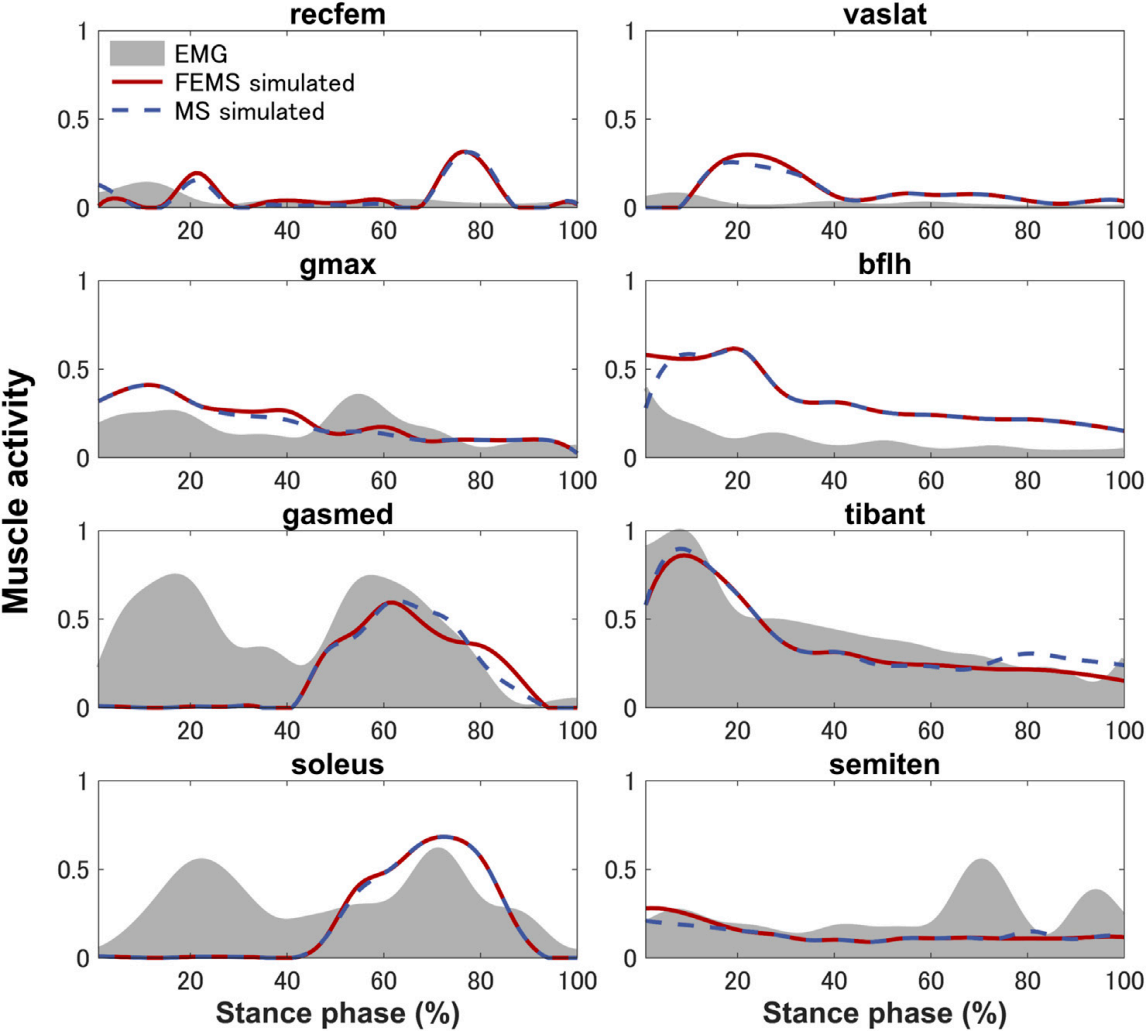
Predicted muscle activities on lower-limb muscles obtained by the finite element musculoskeletal model (FEMS) and the rigid body musculoskeletal model (MS) together with the surface electromyography data recorded from eight muscles during a single stance phase of gait.

The quadriceps femoris muscles (vastus lateralis, vastus medialis, and vastus intermedius), as knee extensor muscles, showed activity mainly with peak activities (0.3, 0.26, and 0.2, respectively) during the loading response, where the rectus femoris is also activated (peaking at 0.32) at approximately 70%–90% of the stance phase (Figure 4) on the FEMS model. The muscle activity of the gluteus maximus was largest (0.41) at 10% of the stance phase. The maximally activated biceps femoris (long head) was 0.61 at 20% of the stance phase. The tibialis anterior activity exhibited a peak trend with a peak activity (0.86) at 10% of the stance phase. The gastrocnemius (medial head) and soleus exhibited similar trends with peak forces (0.59 and 0.68, respectively) later in the stance phase. The similarity between the surface EMG data with the muscle activities calculated from the FEMS and the rigid body MS model was assessed using the root mean square error (RMSE), as shown in Table 2.

**TABLE 2:** Root mean square error (RMSE) values obtained by comparing surface electromyography data with the muscle activities calculated from the finite element musculoskeletal (FEMS) model and the rigid body musculoskeletal (MS) model for the stance phase during gait.

	Recfem	vaslat	gmax	bflh	gasmed	tibant	soleus	semiten
**RMSE** (FEMS)	0.10	0.11	0.21	0.34	0.21	0.29	0.22	0.14
**RMSE** (MS)	0.10	0.10	0.20	0.33	0.22	0.31	0.22	0.13

**FIGURE 5:**
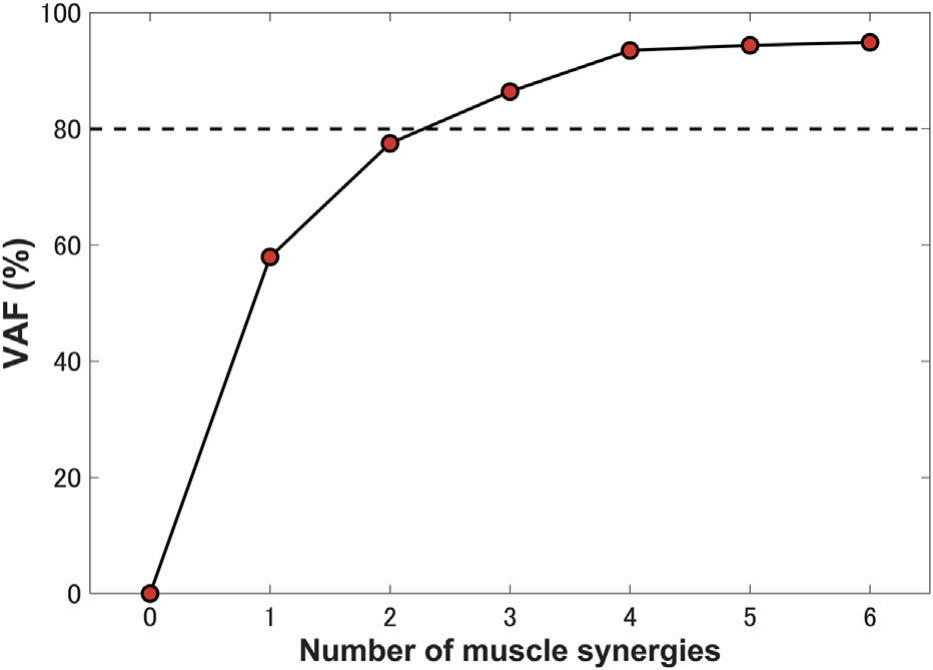
Percentage of variance accounted for (VAF) as a function of the number of extracted synergies. Three synergies were identified for a single stance phase, which accounted for 86.42% VAF.

**FIGURE 6:**
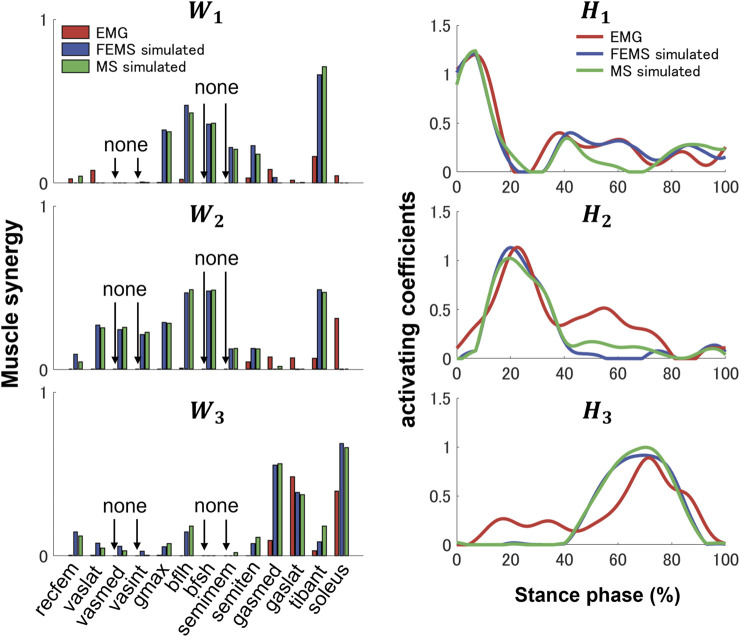
Three muscle synergies, $W_{1}$ , $W_{2}$ , and $W_{3}$ (left-hand panel), and activating coefficients, $H_{1}$ , $H_{2}$ , and $H_{3}$ (right-hand panel), were extracted during the stance phase from 13 main muscles (rectus femoris [recfem], vastus lateralis [vaslat], vastus medialis [vasmed], vastus intermedius [vasint], gluteus maximus [gmax], biceps femoris long head [bflh], biceps femoris short head [bfsh], semimembranosus [semimem], semitendinosus [semiten], gastrocnemius medial head [gasmed], gastrocnemius lateral head [gaslat], tibialis anterior [tibant], and soleus) using the finite element musculoskeletal model and the rigid body musculoskeletal model (FEMS; blue), the rigid body musculoskeletal model (MS; green), and were extracted from nine muscles (rectus femoris, vastus lateralis, biceps femoris long head, gluteus maximus, semitendinosus, gastrocnemius medial and lateral head, tibialis anterior, and soleus) collected by EMG data (red). The EMG signals with the “None” tag were not measured.

Three synergies (86.42% VAF) were required to reconstruct lower-limb muscle activations during the stance phase of gait. Figure 5 shows the muscle synergies decomposed by muscle activities of the lower limb during the stance phase. In Figure 6, the left-hand panels indicate the muscle synergy, and the right-hand panels indicate the coefficients. The muscle synergy $W_{1}$ consisting mainly of the gluteus maximus, hamstrings, and tibialis anterior was activated during the response loading. Moreover, the muscle synergy $W_{2}$ mainly representing the gluteus maximus, quadriceps femoris, hamstrings, and tibialis anterior was activated during mid-stance. The muscle synergy $W_{3}$ consists primarily of the gastrocnemius and soleus. This synergy was activated during the late stance phase. The muscle synergy and activating coefficient extracted from the FEMS and the rigid body MS model demonstrated a strong similarity.

## 4 Discussion

In the present study, a computational model-based approach for predicting subject-specific muscle synergy during gait was proposed. The computational model of the lower limb combined a musculoskeletal dynamics and knee-joint FE analysis in a single concurrent framework that can provide insight into the realistic interaction between the muscle activation and knee-joint tissue deformation was applied. The interaction between the muscle level and joint level that exists *in vivo* is an important relationship that influences the biomechanics of the musculoskeletal system and can only be described in the concurrent framework. Meanwhile, there are physiological high interactions and dependencies between the nervous and musculoskeletal systems, and this neurophysiologically coupled relationship means that the neuromuscular response is controlled through high-precision commands. We hypothesized that the complexity of the representation of muscles and the knee joint in musculoskeletal system modeling is particularly sensitive to the effects of lower extremity muscle activities (Hume et al., 2018). Thus, the modeling complexity is particularly important when decomposing neural controls (muscle synergies) from muscle activities, which requires further validation. The modeling allows for the representation of structures such as joints, ligaments, and muscles in sufficient detail, including complex wrapping, and realistic contact behavior. The complexity of these muscle and joint representations can provide an accurate separation of muscle synergy. Tissue deformation of the hip and ankle joints has little effect on muscle activity (Li, 2021). Thus, it is feasible that the hip and ankle were assumed to be mechanical joints with specific DOFs. However, the effect of tissue deformation of the knee on changes in muscle activity is enormous. This is determined by the structural characteristics of the knee joint. The muscle activities contribute to the knee joint kinematics and could thus change the deformation and translation of the meniscus and cartilages, and the deformed soft tissues could change the knee secondary kinematics, which in turn would seriously affect the muscle length, line of action, and moment arm, thus affecting the redistribution of the muscle activity. The physiological interaction between muscle–level and joint–level described earlier cannot be represented in traditional rigid body MS models but can be effectively implemented in the model we have developed. Static optimization is the core algorithm used in our research to estimate muscle forces, which has the advantages of non-invasiveness and helping to determine the contribution of muscles to movement. However, the proposed method also has some disadvantages. For example, static optimization assumes that muscles generate forces that balance moments acting on the joint, without considering the dynamics of the system, which can result in inaccurate estimates of muscle forces (Lin et al., 2010). Also, static optimization relies on several assumptions, such as the use of a particular optimization criterion and the assumption of a fixed set of muscle parameters, which may not hold true in all cases (Prilutsky and Zatsiorsky, 2010).

The muscle activities are predicted using the FEMS model and rigid body MS model. Comparing the predicted muscle activation patterns during normal gait trials with the average experimental EMG results (Figure 4) shows that both the FEMS and rigid body MS model exhibit relatively good temporal agreement on the muscles, although at certain time points, the predicted activity characteristics by the model differ from the EMG signals. The key characteristics of the EMG of tibialis anterior, gastrocnemius (medial head), and soleus were well predicted (Figure 4). In the loading response phase, the EMGs of these muscles exhibited a significant amount of activity, due to muscular tension caused by individual differences in high heel strike forces at initial contact. In addition, these trends are also often exhibited during treadmill walking and have been confirmed in other studies (Yao et al., 2019; Cherni et al., 2021). However, these trends cannot be reproduced in the model calculation by optimizing muscle activity through the equilibrium in the joint moments obtained from the inverse dynamics. The activities of the semitendinosus as calculated by the model were within approximately one standard deviation of the EMG. The activities of rectus femoris and vastus lateralis were predicted but there was a time lag with respect to the EMG. The similarity in the trends of muscle activity predicted by the FEMS and rigid body MS models suggests that the observed differences are not related to the complexity of joint modeling but rather may be attributed to the algorithms used in static optimization.

**FIGURE 7:**
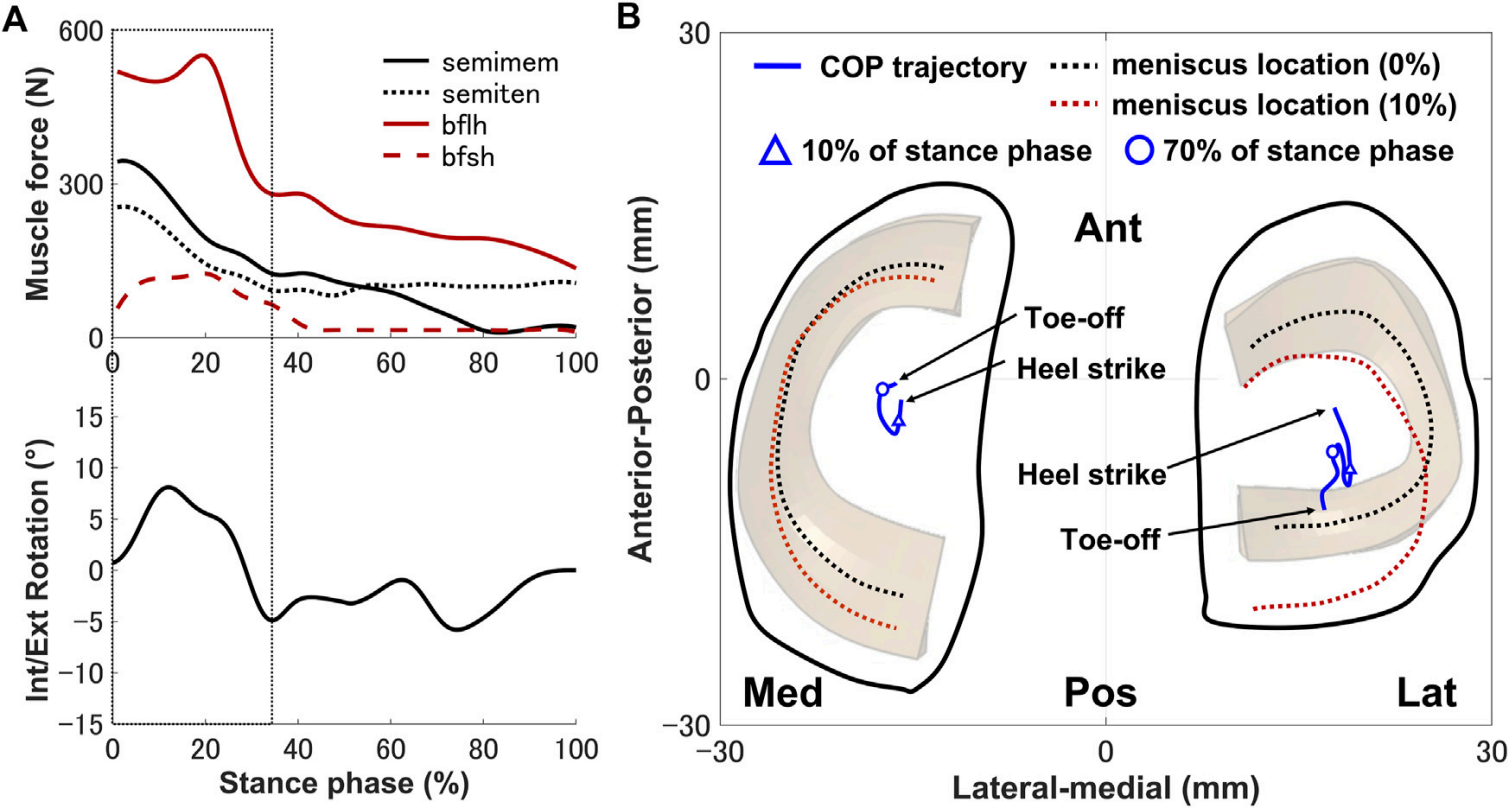
**(A)** Interaction relationship of the hamstrings and the knee rotation under the same stance phase axis. **(B)** Predicted knee contact of pressure (COP) trajectory (blue line) applied to the medial and lateral sides of the tibial cartilage during the stance phase. The dotted lines indicate the meniscal locations at 0% (black) and 10% (red) of the stance phase. The triangle indicates the COP position at 10% of the stance phase, and the hollow circle shows the COP position at 70% of the stance phase.

The secondary kinematics of the knee joint can only be described in the concurrent musculoskeletal framework that represents knee joint motion. However, in order to validate the accuracy of the predicted results, a measurement system capable of directly measuring joint secondary motion is needed for evaluation. Mobile biplane X-ray imaging or dual fluoroscopic imaging technology has been used as the most direct and accurate approach for measuring joint kinematics during human movement (Banks and Hodge, 1996; Mahfouz et al., 2003). Some researchers have demonstrated significant subject-dependent variations in the trend and magnitude of experimental secondary kinematics obtained by mobile imaging (Kozanek et al., 2009; Clément et al., 2018; Gray et al., 2019) (Figure 3). However, the calculated varus–valgus, anterior–posterior, and medial–lateral translations were approximately within the standard deviation of the experimental data, as shown in Figure 3. The knee secondary kinematics can be explained as the combined effect of the muscle forces and contact behavior of joint soft tissues in human locomotion. The internal rotation of the tibia occurred with maximum rotation during the loading response, as shown in Figure 3. The semimembranosus and semitendinosus were activated during the loading response, and the total forces of these muscles acted as an internal rotator of the knee. Moreover, the difference in the medial and lateral structures of the femoral condyle, tibial plateau, and soft tissues leads to a tibial pivot point closer to the medial side than the lateral side, inducing tibial internal rotation (Figure 7A). It can be observed that with knee flexion, the lateral meniscus translates more posteriorly than the medial side at 10% of the stance phase (Figure 7B). The knee remained in the external position without presenting a trend in internal rotation from approximately 60%–70% of the stance phase, which is different from what was observed experimentally. Only the biceps femoris (long head) maintained a certain activity in the component of the hamstrings during this phase, which might explain the discrepancy in the rotation. Afterward, the tibia performed internal rotation until it returned to its initial angle, which is similar to what was observed experimentally during the pre-swing phase. During the pre-swing phase, the hamstrings were not activated and the knee started to flex, whereas the tibia was expected to rotate internally (a reversal of the screw-home movement). The tibial translation occurred with maximum anterior translation during the loading response. The main reason for the tibial translation is that meniscal deformation and translation occurred with accompanying knee flexion, which are crucial functions affecting knee translation (Figure 7B). The secondary kinematics of the knee, especially internal–external rotation and anterior–posterior translation, is an important indicator that influences the motion stability of the knee joint. Moreover, the precise representation of the physiological interaction of the knee joint kinematics and muscle activity could provide an accurate solution for the estimation of muscle synergy.

Previous studies have demonstrated that several muscle synergies control the total patterns of substantial muscular activation on a lower limb during gait (Groote et al., 2014; Saito et al., 2018). Three muscle synergies exceeded 80% of the total VAF for 20 muscles on the lower limbs during the stance phase, which is consistent with results of previous studies (Ivanenko et al., 2005; Esmaeili et al., 2022). Thus, in a single stance phase of gait condition, approximately three muscle synergies controlled the majority of lower-limb neuromuscular activation. Evidence supporting the concept of muscle synergy as modules of locomotion construction comes from research that has examined muscle patterns under a variety of behaviors, including planar covariance of limb joint angles in a cat posture (Lacquaniti et al., 1984; Lacquaniti and Maioli, 1994), flexible combination of spinal modules producing a wide range of movement in a simple manner in frogs (Bizzi et al., 1991; Bizzi et al., 2000), highly similar synergies and patterns of modulation during grasping in monkeys (Overduin et al., 2008), and muscle synergy decomposition in human locomotion (Ivanenko et al., 2005). Of note, researchers found that different factorization algorithms identified similar synergies under different hypotheses and constraints in simulated and experimental data (Tresch et al., 2006). Their results strongly demonstrate that the extracted muscle synergy reflects the basic modules of the human movement system, rather than some computational artifact dependencies on algorithmic choices. Ensuring the consistency between EMG and model simulation regarding spatial and temporal muscle synergy coordination poses challenges due to the limited availability of recorded EMG data compared to the number of muscles in the model. In the present study, we primarily focus on maintaining consistency in the activating coefficients during gait. In order to achieve this, we use the activating coefficients derived from EMG decomposition with NMF as a reference. We verify that the peak values of each decomposed activation coefficient from the model align with the corresponding peak values derived from EMG decomposition. The tibialis anterior controlled by synergies $W_{1}$ and $W_{2}$ is active from heel strike to mid-stance during the stance phase (Figure 6). The tibialis anterior assists dorsiflexion of the foot at the talocrural joint and inverts this dorsiflexion at the subtalar joint. This plays a crucial role in the activities during the first half of the stance phase, which stabilizes the ankle joint as the heel strikes the floor and controls the transition from heel strike to mid-stance. The gluteus maximus also controlled from the two synergies mainly activated during the first half of the stance phase. The gluteus maximus extends the hip joint and simultaneously pulls the pelvis posteriorly. The activation of the muscle occurs in synergy with the same action of the hamstrings represented in synergy $W_{2}$ . This moves the torso from a flexion posture to an upright posture during the mid-stance phase. In addition, the muscle helps maintain an upright posture by balancing the pelvis on the femoral head. The hamstrings are activated simultaneously during the stance phase, which ensures both hip joint extension and knee flexion. Synergy $W_{1}$ controls that the hamstrings flex the knees, and the semimembranosus and semitendinosus, which act as accessory stabilizers of the knee, internally rotate the tibia, acting together with the hamstring muscles and complementing the function of the medial collateral ligament. In synergy $W_{2}$ , biceps femoris are strongly activated and contribute to hip extension and knee flexion. The activation of these muscles helps the torso in bending forward and realizing an upright posture. During this phase, the knee joint is extended, biceps femoris produces tibial external rotation, and the anterior and posterior cruciate ligaments become tangled and tightened. Consequently, the knee joint becomes locked in a position. The tibia becomes stable to ensure the leg upright position during the mid-stance. Furthermore, the hamstrings are activated simultaneously with the quadriceps femoris controlled by synergy $W_{2}$ . This is most important as an antagonist of the quadriceps femoris in the deceleration of knee extension. The rectus femoris is consistently biphasic, also being active at the loading response and the preswing, corresponding to synergies $W_{2}$ and $W_{3}$ . The two phases of rectus femoris activity have entirely different purposes: the first phase provides knee stability during loading response (activating coefficient $H_{2}$ ), and the second phase (activating coefficient $H_{3}$ ) is initiated late in the stance phase and continues during knee joint flexion to assist in hip joint flexion. In synergy $W_{3}$ , the gastrocnemius and soleus are active during the second half of the stance phase. The gastrocnemius is a powerful plantarflexor of the foot, even though it also flexes the knee joint. The function of the soleus is closely related to that of the gastrocnemius muscle. Together, they constitute a chief plantar flexor muscle synergy ( $W_{3}$ ). During the second half of the stance phase, i.e., the start of knee flexion, the plantar flexion attributes of the gastrocnemius are very limited, making the soleus the main muscle responsible for plantar flexion. The plantar flexor muscle synergy, i.e., $W_{3}$ , provides vertical support and has opposite energetic effects on the leg and torso that together ensure support and forward progression of both the leg and torso and accelerate the leg into the swing phase. In summary, synergy $W_{1}$ controls the ankle dorsiflexors to act eccentrically to prevent slapping of the foot on the ground. Moreover, the gluteus maximus and hamstrings extend the hip joint and flex the knee joint in order to ensure that the body weight will be accepted by the leg making contact with ground. Synergy $W_{2}$ controls the tibialis anterior to dorsiflex the ankle, and the gluteus maximus continues hip joint extension. Moreover, quadriceps femoris extended the knee to slow knee flexion by the hamstrings in order to maintain the equilibrium on the lower limb while allowing forward progression. As a plantar flexor muscle synergy, synergy $W_{3}$ produces a propulsive push-off force to advance the extremity into the swing phase. The muscle synergies decomposed from the EMG data were compared with the predicted muscle synergies. Differences in muscle synergies might arise from the different number of muscles in both modeling and EMG data analyses (Steele et al., 2013). Since signal collection for some deep muscle activities, such as those of the vastus medialis and vastus intermedius, was not possible. In the present study, the EMG-based muscle synergies were normalized based on the activating coefficients predicted by the model. We observed that each activating coefficients maintained a high degree of activity consistency at the main peak level. Although it is not possible to compare the muscle synergy, the predictive accuracy of the model can be assessed by qualitatively evaluating the activating coefficient.

The present study may provide evidence suggesting that muscle synergy analysis may be useful for musculoskeletal disorders and focuses on characteristic modular structures in muscle synergy rather than the number of modules. Most recent studies have attempted to apply muscle synergy analysis to neurological diseases through the number of modules to analyze the severity of the disease (Clark et al., 2010). However, it is difficult to identify underlying neurological disorders from changes in modular structure on muscle synergy because patients with neurological disorders exhibit a wide variety of symptoms, regardless of the location of the disease. In contrast, in musculoskeletal disorders, muscle synergy analysis enables extraction of disease-specific modular structures because the phenotypes of musculoskeletal disorders are directly reflected in changes in muscle activity. In addition, musculoskeletal disorders cause various types of abnormal movements with joint dysfunction, for example, knee osteoarthritis. These changes in muscle activity and joint structure may affect muscle synergy. By quantifying the muscle activity contained in the muscle synergy, characteristic modular structures on musculoskeletal disease may provide indication of neuromuscular features and reveal anomalies underlying musculoskeletal disease. The complexity representation of joints in the proposed model offers the possibility of quantitative muscle synergy analysis of joint disorders. In addition, although decomposing muscle synergy through EMG analysis is a widely adopted and accurate method, our goal is to globally understand the mechanism of knee joint diseases by comprehending the impact of joint abnormal movements on muscle synergy. Therefore, it is meaningful and necessary to develop a workflow from musculoskeletal modeling on a finite element framework to muscle synergy analysis.

The present study had some limitations. First, the FEMS lower-limb model and gait data were not from the same subject. The most accurate computational analysis of lower-limb biomechanics requires subject-specific model geometry and human motion data. Second, only one subject participated in the gait measurement experiment. Due to individual differences, the generalizability of our findings may be affected by random individual traits. However, the present study focused on the development of a new approach of using a single finite element framework as a musculoskeletal model to provide a potential tool by which to elucidate the interactions between the nervous system and the joint-level-musculoskeletal system. Third, a different number of muscles in the modeling and EMG data analysis resulted in the impossibility of a quantitative comparison of the decomposed muscle synergy. A final limitation was that the articular cartilages were defined as linear elastic isotropic material, whereas a biphasic fibril-reinforced material might better approximate the representation of the dynamic response of the cartilage (Brindle et al., 2001) and may impact the calculation of the joint kinematics, thereby affecting the back prediction of neural instructions.

## 5 Conclusion

In the present study, a computational musculoskeletal model concurrent knee FE analysis was developed and used to investigate characteristic modular neuromuscular structures on the lower limb using muscle synergy analysis during the stance phase of gait. Our research demonstrated that 20 representative muscles on the lower limb during the stance phase of gait could be described by three simple modules of muscle synergies. These muscle activities are reasonably well described by three synergies, which could reduce the dimensionality of the control problem on the central nervous system. These muscle synergies can explain well the motion mechanism of the lower limbs during gait, because this is consistent with the behavior of the musculoskeletal biomechanics. The modeling may provide a potential tool for understanding the neurophysiologically coupled relationship between the nervous and joint-level musculoskeletal system, such as the investigation of indication of neuromuscular features on knee osteoarthritis and the formational mechanism of joint constriction due to cerebral palsy.

## Data Availability

The original contributions presented in the study are included in the article/supplementary material, further inquiries can be directed to the corresponding authors.
